# IMPRESS-Norway: improving public cancer care by implementing precision medicine in Norway; inclusion rates and preliminary results

**DOI:** 10.2340/1651-226X.2024.28322

**Published:** 2024-05-23

**Authors:** Katarina Puco, Gro Live Fagereng, Sigmund Brabrand, Pitt Niehusmann, Egil Støre Blix, Eli Sihn Samdal Steinskog, Åse Haug, Cecilie Fredvik Torkildsen, Irja Alida Oppedal, Sebastian Meltzer, Åsmund Flobak, Kajsa Anna Margareta Johansson, Line Bjørge, Geir Olav Hjortland, Astrid Dalhaug, Jo-Åsmund Lund, Bjørnar Gilje, Marte Grønlie Cameron, Randi Hovland, Ragnhild S. Falk, Sigbjørn Smeland, Hege Elisabeth Giercksky Russnes, Kjetil Taskén, Åslaug Helland

**Affiliations:** a Institute for Cancer Research, Oslo University Hospital, Oslo, Norway; b Department of Oncology, Oslo University Hospital, Oslo, Norway; c Department of Pathology, Oslo University Hospital, Oslo, Norway; d Department of Oncology, University Hospital of North Norway, Tromsø, Norway; e Department of Oncology and Medical Physics, Haukeland University Hospital, Bergen, Norway; f Department of Obstetrics and Gynecology, Stavanger University Hospital, Stavanger, Norway; g Department of Thoracic Medicine, Haukeland University Hospital, Bergen, Norway; h Department of Oncology, Akershus University Hospital, Lørenskog, Norway; i Department of Oncology, Trondheim University Hospital, Trondheim, Norway; j Department of Obstetrics and Gynecology, Haukeland University Hospital, Bergen, Norway; k Department of Oncology, Nordland Hospital, Bodø, Norway; l Clinic for Cancer Treatment and Rehabilitation, Møre and Romsdal Hospital Trust, Ålesund, Norway; m Department of Hematology and Oncology, Stavanger University Hospital, Stavanger, Norway; n Department of Oncology, Sørlandet Hospital, Kristiansand, Norway; o Department of Cancer Genomics, Haukeland University Hospital, Bergen, Norway; p Oslo Centre for Biostatistics and Epidemiology, Oslo University Hospital, Oslo, Norway; q Division of Cancer Medicine, Oslo University Hospital, Oslo, Norway

**Keywords:** Advanced cancer, targeted therapies, precision cancer medicine, drug repurposing, IMPRESS-Norway

## Abstract

**Background and purpose:**

In Norway, comprehensive molecular tumour profiling is implemented as part of the public healthcare system. A substantial number of tumours harbour potentially targetable molecular alterations. Therapy outcomes may improve if targeted treatments are matched with actionable genomic alterations. In the IMPRESS-Norway trial (NCT04817956), patients are treated with drugs outside the labelled indication based on their tumours molecular profile.

**Patients and methods:**

IMPRESS-Norway is a national, prospective, non-randomised, precision cancer medicine trial, offering treatment to patients with advanced-stage disease, progressing on standard treatment. Comprehensive next-generation sequencing, TruSight Oncology 500, is used for screening. Patients with tumours harbouring molecular alterations with matched targeted therapies available in IMPRESS-Norway, are offered treatment. Currently, 24 drugs are available in the study. Primary study endpoints are percentage of patients offered treatment in the trial, and disease control rate (DCR) defined as complete or partial response or stable disease in evaluable patients at 16 weeks (W16) of treatment. Secondary endpoint presented is DCR in all treated patients.

**Results:**

Between April 2021 and October 2023, 1,167 patients were screened, and an actionable mutation with matching drug was identified for 358 patients. By the data cut off 186 patients have initiated treatment, 170 had a minimum follow-up time of 16 weeks, and 145 also had evaluable disease. In patients with evaluable disease, the DCR was 40% (58/145). Secondary endpoint analysis of DCR in all treated patients, showed DCR of 34% (58/170).

**Interpretation:**

Precision cancer medicine demonstrates encouraging clinical effect in a subset of patients included in the IMPRESS-Norway trial.

## Introduction

Precision medicine is changing oncology by leveraging advanced molecular precision diagnostics, innovative clinical trials, and an expanding panel of targeted drugs and treatment options. Access to adequate molecular diagnostics and drugs is crucial to have an impact and move towards implementation in the national healthcare systems. In Norway, a precision cancer medicine ecosystem has been built in recent years [1]. Next-generation sequencing of tumour tissue and circulating tumour DNA (ctDNA), gene expression profiling, and whole genome sequencing are being implemented in cancer diagnostics worldwide, which has resulted in the identification of a number of specific molecular alterations that drive malignancies. This subsequently enables targeted treatment of specific cancer indications. Although targeted drugs are approved for specific tumour types, the same molecular alterations can also be present in multiple other tumour types, where the efficacy of the treatment is still not tested, typically due to the rarity of the alterations or a low incidence of the tumour type. While an increasing number of new anti-cancer drugs targeting specific molecular alterations enter the market annually, access to these therapies is still unequal. This particularly affects patients with the poorest prognosis who have exhausted all lines of standard-of-care therapies, those with tumours carrying rare mutations, and patients with rare cancers or carcinoma of unknown primary. This is now investigated in pragmatic national clinical trials such as the DRUP trial (Drug Rediscovery Protocol) in the Netherlands [2] and a family of similar trials in several European countries [3, 4] including the IMPRESS-Norway trial [5].

The primary objective of the IMPRESS-Norway trial is to facilitate patient access to commercially available targeted anti-cancer therapies, and to describe anti-tumour activity and toxicity of targeted therapies. Secondary objectives include further analysis of treatment responses and biomarker analysis. Detailed trial design and trial objectives, including the statistical analysis plan, have been published previously [5]. The trial is still recruiting patients, and the final data analysis will be presented at the later time point. This study reports on the primary endpoint of the IMPRESS-Norway trial per October 1, 2023.

## Patients and methods

### Study design and endpoints

IMPRESS-Norway is a national, investigator-initiated, prospective, open label, non-randomised, combined basket and umbrella trial. The trial includes patients with incurable progressing cancer disease with no further standard therapy available. Patients are included into treatment cohorts based on tumour type, molecular alteration, and treatment used. The trial uses a Simon two-stage model for adaptive cohort expansion while minimising the number of patients required [6].

Primary study endpoints are: 1) percentage of the patients included in the trial based on their molecular profile, and 2) disease control rate (DCR) defined as objective complete response (CR), partial response (PR) or stable disease (SD) at 16 weeks (W16) after treatment initiation according to established response criteria.

Secondary study endpoints include supportive efficacy analyses, and in this short report, we also include data on DCR in the whole treated population.

All patients who had completed the molecular screening are used for calculating the percentage of patients included and treated in the trial.

The response evaluable population consists of the subset of patients with measurable disease according to established response criteria, and is used to calculate the DCR at W16 after treatment initiation (primary endpoint). Clinical evaluation of unequivocal progressive disease (PD) was accepted as evaluation method in case of inability to perform radiological evaluation. Patients that stopped treatment due to toxicity, withdrawal or death without PD before W16 evaluation, were excluded from the primary endpoint analysis, while patients with progression as defined by established response criteria prior to W16 were included.

For the secondary endpoint analysis, we performed DCR analysis on all included patients who started treatment without major protocol deviations and who received at least one dose of therapy.

All patients had a minimum follow up time of 16 weeks.

### Patient population and treatment assignment

Adult patients with advanced incurable malignancies, including haematological malignancies, are eligible for inclusion. All patients must meet study defined inclusion and exclusion criteria and sign an informed consent for molecular screening. Second, drug specific informed consent is obtained prior to treatment initiation, based on molecular screening results and alocated treatment, and after progression on all standard anti-cancer treatment. All patients must meet drug-specific inclusion and exclusion criteria and have clinical or radiological progression as assessed by treating physician before treatment start.

Due to limited capacity of molecular profiling, patients with rare cancer types with few treatment options and patients with tumours having an increased probability of finding actionable alterations, had a screening priority at study initiation. However, screening capacity is continuously increasing, and we expect to screen all referred patients by the end of 2024.

The comprehensive molecular profiling of archival tumour tissue is performed using the Illumina TruSight Oncology 500 (TSO500) gene panel, and screening is reimbursed as part of public healthcare in Norway. In addition, ctDNA analysis by Roche FoundationOne Liquid CDx assay (Foundation Medicine, Inc.) was performed in the first 500 screened patients, as well as for patients with no available tumour tissue and where new biopsies could not be collected. Additional diagnostic tests, such as immunohistochemistry (IHC), fluorescence *in situ* hybridisation (FISH), or other molecular/diagnostic tests, can be used to confirm molecular findings. All screened patients are discussed at the Virtual National Molecular Multidisciplinary Tumor Board. If a targetable molecular alteration is identified and a matching trial drug is available, the patient is offered inclusion in the treatment phase of the study. Currently, 24 different drugs are available in IMPRESS-Norway, of which three are available only for haematological malignancies (Supplementary Table 1).

### Efficacy assessments

Patients included in treatment cohorts are evaluated at treatment weeks 8, 16, 26, and every 3 months thereafter. Response is evaluated by RECIST v1.1 [7] in solid tumours, RANO [8] in brain tumours, IWG-ELN, IMWG criteria [9, 10] and CHESON/Lugano recommendations [11] in haematological cancers and non-Hodgkin lymphoma, respectively, and iRECIST [12] is used for immunotherapy evaluation. Patients are receiving treatment until disease progression, unacceptable toxicity, death, consent withdrawal or withdrawal by the decision of the study investigator.

### Data collection and statistical analysis

Data are collected from electronic report case form (eCRF) Viedoc. R version 4.3.2 was used for statistical analysis. Patient characteristics and tumour responses were summarised using descriptive statistics.

## Results

### Patients

IMPRESS-Norway opened for accrual at April 1, 2021. By October 1, 2023, 1,167 patients had completed molecular profiling and subsequent evaluation for inclusion in the study treatment phase. The median age for patients included into molecular screening was 58 years (range 18–84 years), the majority of the patients had Eastern Cooperative Oncology Group (ECOG) Performance Status 0–1, and equal number of female and male patients were included. Patients included in the treatment phase had a median age of 60 years (range 19–80 years), 81% had ECOG 0–1, and 55% were female. The most common cancer types included were colorectal cancer, lung cancer, and cholangiocarcinoma. Baseline patient characteristics of screened patients and patients included in the treatment phase are shown in Supplementary Table 2.

**Figure 1:**
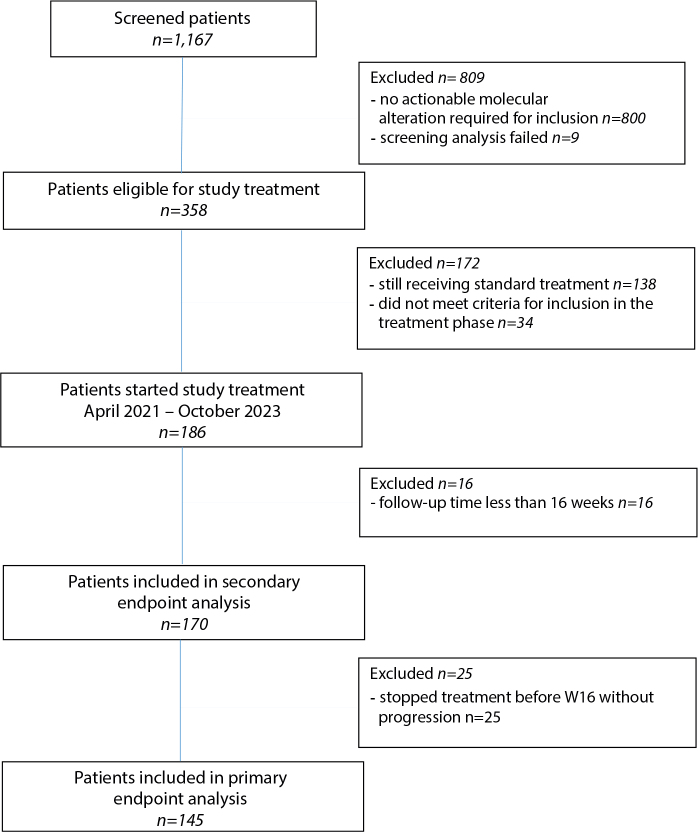
Flow diagram of patients included in the IMPRESS-Norway analysis.

A total of 358 of all screened patients (31%) had an actionable molecular alteration and a matching targeted drug eligible for inclusion in the treatment phase of the study. Of these, 138 patients were still receiving standard treatment and are candidates for inclusion upon progression on standard therapy. Thirty-four patients did not meet the criteria for initiating treatment, commonly due to disease progression and general deterioration of their condition during screening, or they did not meet drug-specific inclusion criteria. By October 2023, 186 patients started treatment, 16 patients had follow up time less than 16 weeks, and 25 patients stopped treatment without detected progression before W16 due to toxicity (*n* = 18), death (*n* = 2) or withdrawal (*n* = 5). Thus, response evaluable population consists of 145 patients. A schematic overview of screened patients and patients included in the treatment phase and efficacy analysis is shown in Figure 1. Patients have been included in 109 different treatment cohorts based on tumour type, genomic alteration, and targeted therapy. A complete list of used therapies and the number of patients treated is available in Table 1.

**Table 1:** Number of patients treated with different treatments/treatment combinations.

Study treatment/treatment combination	Number of patients treated, *n* = 170
Trametinib	35
Pertuzumab and trastuzumab	31
Atezolizumab	25
Cobimetinib and vemurafenib	19
Alpelisib	18
Trametinib and dabrafenib	14
Atezolizumab and bevacizumab	7
Pemigatinib	7
Vismodegib	5
Olaparib	2
Imatinib	2
Alectinib	2
Alpelisib and fulvestrant	1
Entrectinib	1
Capmatinib	1

### Disease control rate at Week 16, preliminary results

The primary endpoint, DCR at W16 was 40% (58/145); 1 patient (<1%) had CR, 17 (12%) patients PR, and 40 patients (28%) had SD. Eighty-seven patients (60%) had PD at W16. Progression was radiologically confirmed in 50 patients, while 37 patients had unequivocal clinical PD.

The secondary endpoint, DCR at W16 in all treated patients was observed in 34% (58/170) of the patients.

**Figure 2:**
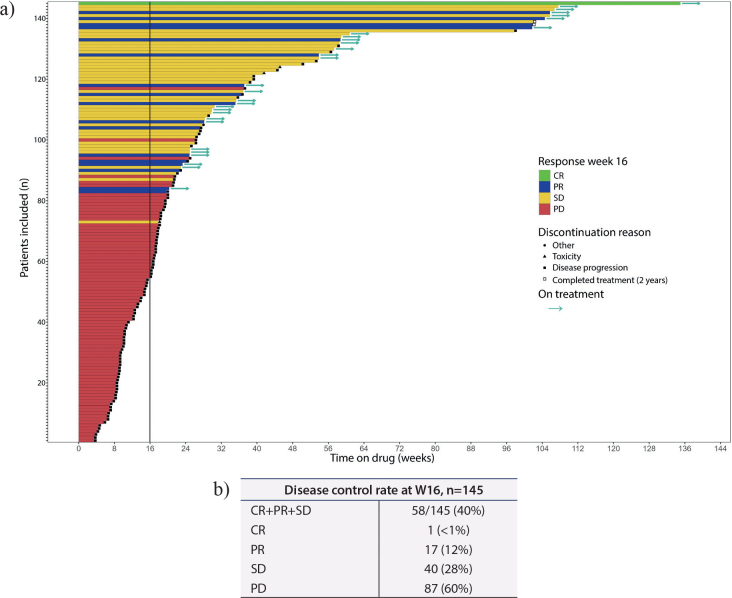
Preliminary results. a) Swimmer plot of the time on treatment, observed response at W16, and reason for treatment stop in response evaluable population, n=145. b) Disease control rate among response evaluable patients at W16, n=145

Preliminary results are summarised in Figure 2.

## Discussion

Preliminary results from the IMPRESS-Norway trial show that comprehensive molecular tumour profiling is feasible and confirm the presence of targetable molecular alterations, leading to additional experimental targeted treatment in around 30–40% of all screened patients, either as a treatment in IMPRESS-Norway, other studies or early access programmes. This is in accordance with earlier published meta-analysis and other similar studies, where the percentage of patients having targetable alterations varied from 30 to 88% [13–15].

The observed DCR at 16 weeks of treatment for evaluable patients, was 40%. The first European precision medicine trial, SHIVA, reported no clinical benefit in 99 treated patients in 2015 [16], the MOSCATO 01 trial reported objective response rates of 11% [17]. More recently, the DRUP trial reported clinical benefit rate at 16 weeks of 34% [2], whereas the CoPPO trial in Denmark and first results from the MyPathway trial in the United States reported objective response rates in 15 and 23% of treated patients, respectively [18, 19].

IMPRESS-Norway, like other precision medicine trials, has limitations that need to be taken into consideration when interpreting results. The majority of the trials are non-randomised lacking a control group. There are several differences between the precision medicine trials, for example, molecular profiling tests used for inclusion, changes in understanding and interpretation of molecular findings, and access to targeted treatments over time. Study endpoints varied from progression free survival, response rates, clinical benefit and DCR. Therefore, comparison and the interpretation of results may be difficult. However, our results seem to be in line with results reported in later trials.

Due to limited availability of molecular profiling and access to drugs at IMPRESS-Norway initiation in 2021, patients with an increased probability of finding targetable alterations were prioritised for screening, indicating a certain degree of patient pre-selection to the trial. This could lead to a higher percentage of actionable findings than if an unselected population was screened. In some of the other studies, like the DRUP trial, all patients were pre-selected prior to referral for inclusion. On the other hand, in IMPRESS-Norway, targetable alterations were defined by the availability of matching drug, meaning potentially targetable alterations that were not acted upon, were not counted as actionable, indicating that our reported percentage could be higher. As the larger gene-panels are becoming more available and a number of targeted therapies is increasing, it is expected that a higher proportion of patients will have actionable targets within the study.

In conclusion, the introduction of national comprehensive molecular diagnostics has ensured additional treatment options for approximately one-third of patients screened in IMPRESS-Norway trial. Increased knowledge on molecular targets, access to comprehensive molecular diagnostics, and targeted treatments contributed to the observed increased benefit compared to the first reported precision medicine trials.

IMPRESS-Norway continues to recruit patients and collaborates with other DRUP-like clinical trials across Europe, such as the ProTarget trial in Denmark [3] and FINPROVE in Finland [4]. Through the European initiatives PCM4EU [20] and PRIME-ROSE [21], DRUP-like clinical trials have built a distributed clinical trial network that addresses national priorities while collaborating internationally for scale and impact.

## Authors contribution

GLF, ESB, ÅF, J-ÅL, RH, SS, HEGR, KT, ÅH, BTG have provided study concept and design. KP, GLF and ÅH have provided manuscript draft. KP, GLF, ÅH and RSF have analysed and interpreted data. All authors have contributed with data acquisition and critical revision of the manuscript.

## Supplementary Material



## Data Availability

The full clinical dataset consists of de-identified patient-level data obtained from VieDoc. The sponsor and data owner is Oslo University Hospital. Access to full raw patient-level data is limited, but project partners can apply for access through the data and biobank committee of the trial, in accordance with Data Privacy and Ethical Approval for the study project. All authors have full access to complete study data, study analysis performed, tables and figures. The study protocol, including a statistical analysis plan, is available.
