# Supplementary material for: IMPRESS-Norway: improving public cancer care by implementing precision medicine in Norway; inclusion rates and preliminary results
Source: Acta Oncol. 2024 May 23;63:28322. doi: 10.2340/1651-226X.2024.28322 (PMC11332498; doi:10.2340/1651-226X.2024.28322)
Supplement: Supplementary file 1 [file AO-63-28322-s1.pdf]

Supplementary material has been published as submitted. It has not been copyedited, or typeset by Acta Oncologica

Supplementary Table 1. Treatments available in IMPRESS-Norway

Drug name	Mechanism of action	Available since*	Company
**Alectinib**	ALK and RET inhibitor	April 2021	Roche
**Atezolizumab**	PD-L1 inhibitor	April 2021	Roche
**Bevacizumab**	VEGFR inhibitor	April 2021	Roche
**Cobimetinib**	MEK inhibitor	April 2021	Roche
**Entrectinib**	ALK, ROS-1 and NTRK inhibitor	April 2021	Roche
**Trastuzumab and pertuzumab**	Her-2 inhibitor	April 2021	Roche
**Vemurafenib**	BRAF inhibitor	April 2021	Roche
**Vismodegib**	Hedgehog pathway inhibitor	April 2021	Roche
**Dabrafenib**	BRAF inhibitor	January 2022	Novartis
**Trametinib**	MEK inhibitor	January 2022	Novartis
**Alpelisib**	PI3K inhibitor	June 2022	Novartis
**Fulvestrant**	Estrogen receptor inhibitor	June 2022	Novartis
**Olaparib**	PARP inhibitor	June 2022	Astra Zeneca
**Imatinib**	Abl, c-Kit and PDGFR inhibitor	June 2022	Generic
**Capmatinib**	MET inhibitor	November 2022	Novartis
**Pemigatinib**	FGFR inhibitor	February 2023	Incyte
**Selpercatinib**	RET inhibitor	February 2023	Eli Lilly
**Bortezomib**	Proteasome inhibitor	February 2023	Generic
**Tepotinib**	MET inhibitor	March 2023	Merck
**Melphalan**	Alkylating agent	June 2023	Generic
**Actinomycin D and hydroxyurea**	Cytotoxic antibiotic and antimetabolite	August 2023	Generic
**Ceritinib**	ALK inhibitor	Expected December 2023	Generic
**Dostarlimab**	PD-1 receptor inhibitor	Expected December 2023	GSK
**Niraparib**	PARP inhibitor	Expected December 2023	GSK

*Available since refers to time point from which eligible patients could start treatment on the drug.

Supplementary Table 2. Baseline characteristics of the patients included in screening and treatment phase of the study

	**Screening phase (n=1,167)**	**Treatment phase (n=170)**
**Age in years, median (range)**	58 (18-84)	60 (19-79)
**Gender, n (%)**		
**Male**	572 (49%)	76 (45%)
**Female**	595 (51%)	94 (55%)
**ECOG performance status, n (%)**		
**ECOG 0**	371 (32%)	61 (36%)
**ECOG 1**	416 (36%)	78 (45%)
**ECOG 2**	130 (11%)	30 (18 %)
**Unknown***	250 (21%)	1 (<1%)
**Tumor types, n (%)**		
**Colorectal Cancer**	154 (13%)	16 (9%)
**Cholangiocarcinoma**	83 (7%)	18 (11%)
**NSCLC****	78 (7%)	14 (8%)
**Breast Cancer**	72 (6%)	4 (2%)
**GBM***, IDH wildtype**	53 (5%)	9 (5%)
**Ovarian carcinoma**	46 (4%)	10 (6%)
**Thyroid carcinoma**	21 (2%)	8 (5%)
**Bladder**	19 (2%)	6 (4%)
**Other**	641 (54%)	85 (50%)

* Registration in in electronic case report form (eCRF) is ongoing

** Non-small cell lung cancer

*** Glioblastoma
